# Older and younger adults’ perceptions of augmented reality photorealistic avatars as a viable medium for interpersonal communication

**DOI:** 10.1093/geroni/igaf083

**Published:** 2025-08-09

**Authors:** Mahrukh Tauseef, Akshith Ullal, Alexandra Watkins, Mary S Dietrich, Cathy Maxwell, Judith Tate, Lisa Juckett, Lorraine C Mion, Nilanjan Sarkar

**Affiliations:** Department of Electrical and Computer Engineering, Vanderbilt University, Nashville, TN, United States; Intelligent Clinical Care Center, University of Florida, Gainesville, FL, United States; Department of Mechanical Engineering, Vanderbilt University, Nashville, TN, United States; Department of Biostatistics, School of Medicine and Nursing, Vanderbilt University, Nashville, TN, United States; College of Nursing, University of Utah, Salt Lake City, UT, United States; College of Nursing, Ohio State University, Columbus, OH, United States; School of Health and Rehabilitation Sciences, College of Medicine, Ohio State University, Columbus, OH, United States; School of Health and Rehabilitation Sciences, College of Medicine, Ohio State University, Columbus, OH, United States; Department of Electrical and Computer Engineering, Vanderbilt University, Nashville, TN, United States; Department of Mechanical Engineering, Vanderbilt University, Nashville, TN, United States

**Keywords:** Emotion recognition, Telepresence, Social presence, Human-computer interaction

## Abstract

**Background and Objectives:**

Augmented reality (AR) telepresence is a novel interactive communication modality that maps a user’s 3D photorealistic avatar to another user’s physical environment. However, AR’s application with older adult populations is understudied. As such, we examined young and older adults’ perceptions of utilizing this modality for social communication. Additionally, we tested the participants’ ability to recognize 6 common emotions displayed by 3D photorealistic avatars compared to video clips of real people, examining whether age differences existed in communication perceptions and emotion recognition accuracy.

**Research Design and Methods:**

To assess participants’ perceptions, older (*n* = 31) and younger (*n* = 31) adults interacted with a volunteer’s 3D photorealistic avatar using a structured conversational activity through a head-mounted display (HMD). Participants rated the quality of the HMD-AR communication based on physical and human realism, comfort while talking to the avatar, and the degree of social presence offered by the HMD-AR. Participants then identified 6 basic emotions exhibited by (1) video clips of a real person and (2) an animation of their 3D photorealistic avatars via an HMD. Each participant viewed a total of 36 *video and avatar* stimuli. Subgroup analyses were conducted by age group.

**Results:**

Participants reported a positive communication experience with the 3D photorealistic avatar, with older adults rating the quality higher. Ratings were generally lowest for how life-like the model appeared (68% younger adults; 61% older adults). Most younger participants (93%) were able to accurately identify nonverbal emotions displayed by the avatar; older adults had less overall accuracy (80%).

**Discussion and Implications:**

Participants, including older adults, were enthusiastic regarding AR telepresence for interpersonal communication. Most were able to accurately identify emotions displayed by the 3D photorealistic avatars, although younger adults outperformed older adults. Further technology development will likely enhance the quality of AR communication for everyday use.

Translational Significance Addressing loneliness and social isolation among older adults through interactive communication modalities (ICMs) requires technology that is reasonable in cost and can mirror the communication qualities of in-person visits. Readily available mobile applications can create 3D photorealistic avatars that are acceptable to older and younger adults using head-mounted display augmented reality (HMD-AR). Overall accuracy of identifying non-verbal emotions displayed by the photorealistic avatars was high among both groups, although older adults were less accurate. Further development of HMD-AR is directed toward enhancing its capability as an ICM when in-person visits are not feasible.

## Background and objectives

Interpersonal communication is fundamental in facilitating social connections through the exchange of ideas, emotions, facts, and opinions.^1^ Until recently, interpersonal communication has occurred primarily through direct face-to-face interactions, using spoken language and nonverbal cues to express thoughts and emotions. Nonverbal communication is especially important in expressing one’s affective state and includes explicit cues such as eye gaze and facial expressions, and subtler, yet equally influential, elements of bodily actions like hand gestures and personal space proxemics.^2^ Face-to-face interaction encompasses all these behaviors and is the preferred way of interaction by most people.^3^

### Loneliness and social isolation are common among older adults

Social connection is critical in preventing social isolation and loneliness.^4^^,^^5^ Older adults often face challenges related to traveling for in-person visits with friends and family, such as reduced mobility, retirement relocation, and loss of loved ones. Thus, older adults have high rates of loneliness and social isolation with adverse health consequences, including mortality, cardiovascular disease, depression, suicide, cognitive and physical decline, reduced quality of life, and increased healthcare utilization.^4^^,^^5^ Interactive communication modality (ICM) technology is a potential means to mitigate loneliness and social isolation.^5^

### Social presence as the underlying mechanism in effective ICM technology

Studies of ICM interventions, such as social media and ­video-mediated visits, have shown mixed effects on social connection, social isolation, and/or loneliness among older adults.^6^^,^^7^ The varying designs, ICM mediums, settings, communication partner (eg, family, volunteer), measures, and outcomes make it difficult to draw firm conclusions. One potential explanation for ICM’s mixed results is a lack of attention to social presence within the ICM. *Social presence theory* refers to the ICM quality and the participant’s perceptions, behaviors, or attitudes that other people are physically present and of being present with the “real” person.^8^ Social presence is a transient state that varies with the ICM, knowledge of the other person, content, environment, and context.^8–10^ Satisfaction with ICMs is based largely on the quality of the social presence afforded. Existing ICMs, such as video conferencing (eg, FaceTime), lack elements of face-to-face interactions, including the 3D nature and life-sized proportionality that enable users to experience each other’s presence; thus, ICMs are often regarded as a secondary source of communication, only to be used when face-to-face interaction is not possible.^3^ With its foundation in theories of interpersonal communication and symbolic ­interactionism, social presence is strongly correlated with the awareness of others and with a sense of connection with others.^8^ There is a critical need to design ICMs as primary sources of interpersonal communication comparable to face-to-face interactions, especially for older adults.^10^

### Augmented reality-based telepresence

Head-mounted display (HMD) augmented reality (HMD-AR) is chief among the newer ICMs. HMD technology places digital representations of distant users, that is, avatars, into the physical environment of a local user with the aim of evoking similar emotional engagement and connectedness as face-to-face communication.^11^ Commercially available HMDs for AR (eg, Vision Pro and Meta Quest) overlay 3D models onto the real-world environment, allowing users to see and interact with virtual objects as if they were physically present. HMDs can track nonverbal features, such as a user’s eye gaze or the position and orientation of body parts, enabling users to convey their real body posture and gestures while communicating. Nonverbal components of communication are conveyed through photorealistic 3D avatars, as the body and facial muscles of these avatars can be driven remotely to mimic users’ motion and facial expressions.^12^ These HMDs also have robust networking capabilities to facilitate high-speed transmission of body joint information, facial movement, video, and audio feeds. This allows multiple users to interact with one another through a shared environment from different geographic locations, a phenomenon known as telepresence.^13^

#### Older adults’ use of HMD-AR

Research into HMD-AR applications for older adults is in its early stages. Study designs are primarily non-experimental, consist of small sample sizes, focus on technology hardware and/or software development, and are limited in activities, such as designing a park.^14^ Early studies reveal the promise of HMD-AR as most older adults accepted the technology and experienced few side effects. Suggestions for improvement included: HMD type (lighter weight and fit over eyeglasses); avatar designed to be more realistic; increased size of synthetic objects; enhanced training; and providing different levels of difficulty to address varying physical or cognitive limitations.

#### 3D photorealistic avatars

For telepresence to be effective, it is imperative that the avatar accurately mimics the real person’s bodily actions and facial expressions so that the emotion, meaning, and intent behind them are preserved during remote interaction. Highly detailed 3D photorealistic avatars have traditionally been created using sophisticated camera rigs, such as those used for generating Meta’s codec avatars and Microsoft’s Holoportation.^15^^,^^16^ These technologies capture multiple images from various angles to create a detailed 3D model of a person, which can then be transformed into a realistic avatar. The process is expensive, time-consuming, and requires the user to wear fiducial markers and be recorded with multi-camera systems.^15^^,^^17^ Recent advancements in computer vision and machine learning have enabled the creation of 3D photorealistic avatars using more accessible devices like webcams or smartphone apps, such as in 3D and Polycam. These methods offer a quicker and cheaper alternative to the traditional camera rig systems but sacrifice realism.^18^^,^^19^ While the resulting avatars may not be as photorealistic as those produced by state-of-the-art methods, they provide an important trade-off between resource efficiency and quality.

#### Quality of interpersonal communication with a 3D photorealistic avatar

In-person interactions allow one to feel the presence of others.^3^ Several AR studies, consisting of mostly younger adults, have explored different aspects of in-person interactions through photorealistic avatars, including social interaction, collaborative task performance, and teleconsultation.^20–26^ Key findings were a preference for full-body photorealistic avatars; avatar mannerisms that represented emotion strengthened social connection; mapping facial expressions with body gestures improved communication; and real-time mapping of emotions from user to avatar enhanced social presence. For some, avatar facial expressions evoked levels of uncanniness, that is, discomfort or even revulsion when an avatar looks almost but not quite human-like.^20^

#### Expression of emotions through photorealistic 3D avatars

Non-verbal cues, including emotional expression, are critical in interpersonal communication and essential for social connectedness.^27^ The extent to which 3D photorealistic avatars can convey emotions through HMD-AR is largely unknown. Research on determining humans’ ability to detect emotions expressed by an avatar has focused primarily on facial emotion recognition of an avatar on a 2D screen using video or still images.^28^^,^^29^ Researchers who utilized full-body pose in emotion recognition studies relied on the perception of real-life images and videos, including the use of intelligent algorithms to classify emotions based on body posture.^28–30^

### Gaps in knowledge

Most studies on social presence in HMDs and avatars focused on virtual reality (VR) rather than augmented reality.^9^^,^^31^ One cannot assume that findings from VR devices can be transferred to AR devices, given the solely virtual world of VR vs the mixed reality created by AR. Prior HMD-AR studies focused on 2D rather than 3D photorealistic avatar representations. As HMD-AR technology advances and cost-effective products, including the production of 3D photorealistic avatars, become available, its use as a potential ICM for all age groups is likely. However, few studies involving photorealistic avatars have included older adults, a major shortcoming since age can impact acceptance and use of ICMs^32^ and the ability to accurately identify emotions from facial expressions.^33^ Prior studies lacked a comparison of real-life interactions to photorealistic avatar interactions that would allow a more representative comparison. Research to determine whether AR telepresence can effectively substitute in-person interactions with minimal loss of fidelity requires evaluation of factors affecting the quality of interpersonal communication, including the interaction’s realism, the users’ comfort level, and their ability to interpret each other’s behaviors.

Given the dearth of knowledge on how young and older adults would judge the use of HMD-AR with lower-quality but easier-to-generate 3D photorealistic avatars as a communication medium, the focus of this research was to explore the extent to which it conveyed social presence. The primary aim was to examine the quality of interpersonal communication with a 3D photorealistic avatar created by easily accessible software. Our second aim was to examine the accuracy of emotions projected by these 3D photorealistic avatars as compared to video clips of humans. Because age-related differences may exist in accepting ICM as well as in identifying facial expressions of basic emotions, we examined age-related differences.

## Research design and methods

### Design overview

We used a 2-group within- and between-subjects design. Young adults ranged from 18 to 35 years of age; older adults were 65 years and older. Participants from both groups underwent the same protocols. Baseline questionnaires gathered demographic information and prior experience with HMD-AR. Participants interacted in person with a volunteer, followed by an interaction with the volunteer’s 3D photorealistic avatar (see Figure S1) using a structured conversational activity. Participants then identified emotions displayed by video clips on a computer screen of a real person (*video stimuli*) and animation of their 3D photorealistic avatars (*avatar stimuli*) as seen through an HMD. The 2 activities took approximately 45 minutes, with each activity taking approximately 20 minutes. For older adults, a cognitive screening tool took an additional 15 minutes. At the conclusion of the activities, participants completed a survey that ascertained their comfort, perception, and social presence of the HMD-AR when interacting with the 3D photorealistic avatar. The study protocol was reviewed and approved by the Vanderbilt University Institutional Review Board (IRB Protocol #221944), and participants provided informed consent prior to data collection and study experiments.

### Conceptual framework

Two frameworks guided the study. Social Presence Theory postulates that the ICM property and the person’s perceptions that other people are physically present in the ICM affect the quality of the interaction.^8^ The Unified Theory of Acceptance and Use of Technology (UTAUT) framework postulates that individual characteristics such as age and cognition can impact the acceptance and use of ICM.^32^

### Setting and participants

The study took place at the Vanderbilt University School of Engineering. Two groups were recruited: those 18 to 35 years (young adults; *n* = 31) and those 65 years and older (older adults; *n* = 31). Recruitment strategies included posting flyers on campus, posting on social media, and word of mouth. Eligibility criteria included corrected-to-normal vision, no history of VR/AR sickness or motion sickness, and being physically and cognitively able to participate in the procedures. We assessed older adults’ cognition with the Self-Administered Gerocognitive Examination (SAGE)^34^; this validated screening tool assesses cognition in 5 domains: language, reasoning/computation, visuospatial, executive, and memory and orientation. A score of 17 to 22 (maximum) suggests normal cognition. Those whose score fell below 17 were excluded.

### AR telepresence system architecture

**Figure 1.**
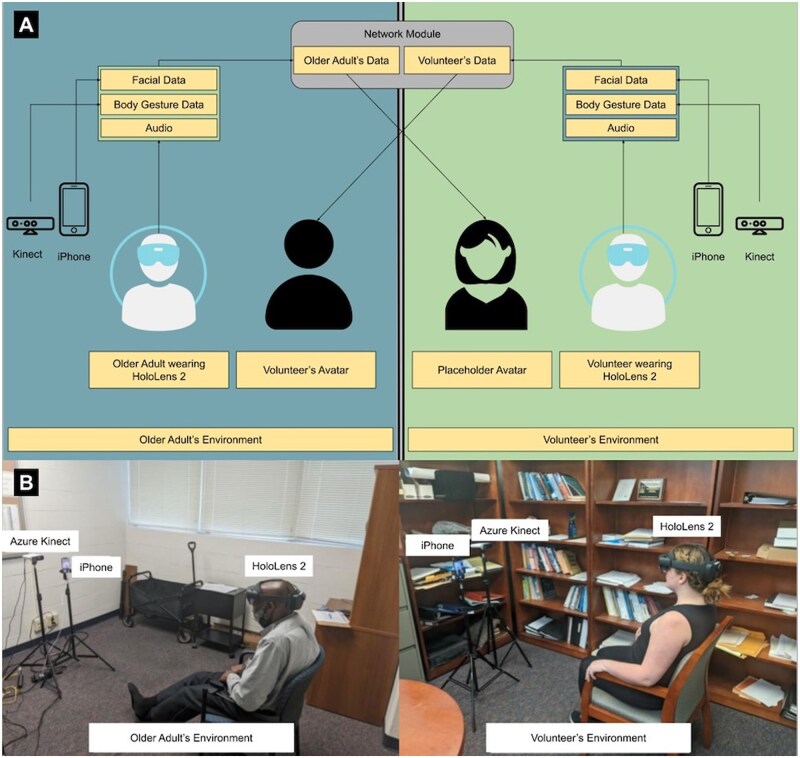
System architecture and study setup. Image A. System architecture used for the head-mounted display augmented reality telepresence experiments that consisted of symmetrical remote and local components with bidirectional communication. Image B shows an older participant conversing with a volunteer through the Augmented Reality setup.

The AR telepresence system (Figure 1A) consisted of symmetrical remote and local components with bidirectional communication. We used the Unity game engine software to build the application for Microsoft HoloLens 2, our choice for the HMD. The application consisted of 2 instances for each user (the volunteer and the participant) with a Photon Unity Voice Networking (PUN) plugin to establish audio communication between them. The body gesture and facial expressions data captured by the Azure Kinect camera and iPhone 12 were transferred across the network using the ZeroMQ (ZMQ) messaging platform (see Supplementary Method in Supplementary Material for further description of the system).

### Perceptions of interpersonal communication with 3D photorealistic avatar

Each participant was introduced to the volunteer in person to allow a comparison between the in-person interaction with the 3D photorealistic avatar interaction they would later participate. The in-person interaction took 5-10 minutes, consisting of the volunteer’s introduction of themselves (eg, engineer student, research staff), asking the participant about themselves (eg, interests), and ending with an expression of gratitude for their willingness to participate. The volunteer proceeded to the remote room that was set up across the hall. A research assistant on the participant’s side coordinated with the volunteer to configure the system and begin the study. Once the system was set up, the participant wore the HoloLens 2 to visualize the photorealistic avatar of the volunteer that mirrored their gestures and facial expressions while synchronously hearing their voice (Figure 1B). For the volunteer, we used a placeholder avatar to represent the participant’s facial expressions and body gestures to allow the volunteer to respond appropriately during the conversation.

For the HMD-AR interaction, we used the “rose, bud, and thorn” activity to allow for a natural and consistent conversation between all participants and volunteers. This activity is often used as a conversation starter and has been used in research studies to stimulate interpersonal communication between participants.^35^ The participants shared one highlight (rose) and one challenge (thorn) from the prior week, and one thing they were looking forward to (bud) in the following week.

Participants rated the quality of HMD-AR interpersonal communication using a 7-item Likert questionnaire (see Figure S3). Using a horizontal orientation for the survey items, each item had a 7-point response with values 1 through 7; only the 2 anchor values had labels: 1 = strongly disagree and 7 = strongly agree. Participants were instructed to choose the numerical value that best reflected their perception. Given the lack of standardized tools to measure the quality of interpersonal communication through HMD-AR telepresence, we generated items using a combination of sources measuring different aspects of AR telepresence. Three statements highlighted physical and human realism as essential aspects of co-presence.^36^ One item measured the participants’ comfort while talking to the avatar given the potential for an uncanny valley effect.^37^ Three statements on mutual understanding and attentional allocation were adapted from the Networked Minds Scale used for measuring social presence in communication technology.^38^ Last, participants provided one word to describe their impression of the experience.

### Non-verbal emotion recognition

Each participant identified 6 basic emotions conveyed by (1) a 3D photorealistic avatar in the HMD-AR telepresence system and (2) video clips of the real person displayed on a desktop computer. We used pre-recorded videos of the volunteers and their 3D photorealistic avatars’ animations to maintain consistency and uniformity. Volunteers were trained to display non-verbal emotions using the Facial Action Coding System (FACS) guideline that categorizes physical expression of emotions through facial movements^39^ and the Bodily Expressive Action Stimulus Test (BEAST)^40^ for expressing emotions and non-verbal communication from body movements.

**Figure 2.**
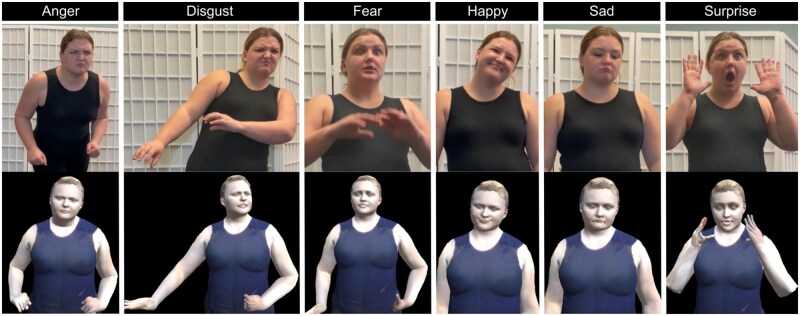
Snapshots of 6 basic emotions validated by an expert in facial and body expressions. The top row consists of video clips. The bottom row consists of the photorealistic avatar.

During the recording, volunteers enacted facial expressions and body gestures for each emotion (anger, disgust, fear, happy, sad, surprise) multiple times, starting from 5 seconds of neutral body pose and facial expression, followed by 5 seconds of the requested emotion, and then another 5 seconds of neutral expression. We recorded the volunteers’ videos and their 3D animations simultaneously and captured their full-body poses and facial expressions. We mapped the recorded 3D animations onto their photorealistic avatars. We sent randomly shuffled videos of the volunteers and their animated avatars to a FACS-certified body-language expert. The expert labeled the videos with the depicted emotion and provided a 3-point confidence rating (low, medium, high). A set of 18 animations (3 animations per emotion) and 18 videos (3 videos per emotion) was shortlisted from the video recordings that the expert labeled correctly with high confidence. Figure 2 shows snapshots of validated video clips and avatar animations of one of the volunteers.

Participants were shown (1) the 18 validated avatar animations through the HMD-AR, and (2) the 18 videos of the real person on a desktop screen. All animations were mapped onto the photorealistic avatar of the same volunteer interacting with a given participant throughout the study. This allowed us to determine the quality of the 3D photorealistic avatar in ­displaying the emotion compared to its human counterpart. Each participant viewed a total of 36 *video* or *avatar* stimuli (3 samples of each of the 6 emotions in both the video clips and the avatar animation formats).

Because presentation of the stimuli differed (either desktop screen for the videos or through the HMD for the avatar animations), each set of stimuli was presented as a block. To mitigate ordering effects, participants were randomly divided into equal groups. One group viewed the avatar animations first (through the HMD), and the other viewed the videos first (on a desktop screen). Presentation of the emotions was randomly ordered for each participant. For example, a participant could have seen “sad,” “happy,” “happy,” “surprised,” “happy,” and so on. We informed all participants that the emotions would appear at random, and any emotion could occur any number of times; we did not inform them that each emotion would occur only 3 times. We provided a written list of the 6 emotions. Individual videos and animations were repeated until the participant determined the emotion prior to moving on to the next video or animation. Participants announced their guess out loud, which was recorded by a research assistant. We did not provide participants with feedback regarding the accuracy of their guesses.

### Data analysis and power

*IBM SPSS Statistics* (version 29) was used for descriptive summaries of the emotion recognition task and the participants’ perceptions of the avatar. We used Mann–Whitney tests (due to ordinal responses) to compare the perceptions of older and younger participants. An alpha of *p *< .05 was maintained and used to interpret statistical significance.

The unit of analysis for emotion recognition accuracy was at the level of each presentation of a given emotion [0 (“Incorrect”) or 1 (“Correct”)]. Each participant (within-subjects factor) had 36 data points for analysis (3 replicates of each of the 6 emotions under each of the 2 study conditions—*video* and *avatar*). The between-subjects factor was the age group (older, younger). We used *Stata* (version 18) to conduct a generalized linear mixed-­effects analysis of our study data. Robust sandwich estimation within the model adjusted the standard error correlations among the within-subjects’ responses while enabling us to analyze the data at the individual emotion presentation response level (a total of 2232 responses; 1116 from the older group and 1116 from the younger group). Thus, within this model, we tested the main and interactive effects of stimulus type (video, avatar), emotion (anger, disgust, fear, happy, sad, surprise), and age (younger adult, older adult) simultaneously on the accuracy with which emotions were perceived. By doing so, we maintained our analysis-wide Type I error rate at 0.05. We estimated marginal mean differences in accuracy between study conditions using a Bonferroni-corrected alpha from within the model to ease the interpretation of the results. Using the specific response to each emotion presentation allowed us to control for a potential ordering of condition effect in the model and sufficient degrees of freedom to analyze differences in recognition accuracy among emotions. A sensitivity analysis (mixed-factor ANOVA) was conducted that used the participant aggregate percentage of correct recognitions for each emotion as the unit of analysis.

Given this was the first work in this area, we used a medium effect size of *d *= 0.5 (or *f *= 0.25) for our detectable effect of interest (differences in emotion recognition accuracy between video and avatar conditions, with a consideration of a potential interaction effect with age group). While we used a much more powerful analytic approach than a mixed-factor ANOVA (within effects: study condition and emotion, between effects: age group), we used the ANOVA approach to conservatively estimate sample size. A total sample of 60 participants (30 younger, 30 older) sufficed to detect a difference as small as 0.5 *SD* in percent accuracy between any 2 cells with a significance level (α) of 0.05, and the standard assumption of 80% power.

## Results

### Participants

Thirty-one younger adults (*n *= 9, 29% female) aged 18-35 years (*M *= 25.8, *SD *= 3.7) and 31 older adults (*n* = 23, 74% female) aged 65 years and above (*M *= 71.0, *SD *= 6.0) constituted the participant sample. Young adults were Asian/Asian-American (71%) or White (29%); none were Hispanic. Older adults were White (55%), African American/Black (29%), or Asian (7%); 9% refused to provide the information; 3% were Hispanic. All participants were asked whether they had prior experience with wearing HMD-AR. While most of the younger adults had experience with HMDs for virtual reality, which are different from head-mounted displays for augmented reality, only 2 indicated they had experience with HMD-AR. None of the older adults had experience with HMD-AR.

### Quality of HMD-AR interpersonal communication

**Figure 3.**
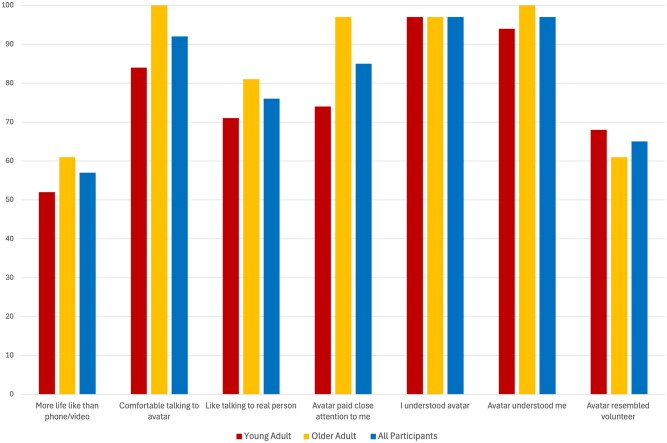
Results on participants’ perceptions of the quality of the interaction with the photorealistic avatar.

Scores 5 or higher on the 7-point Likert scale indicated a positive perception, with the highest proportion of all 62 participants indicating understanding the avatar (*n* = 60, 97%), feeling that the avatar understood the participant (*n* = 60, 97%), and being comfortable in talking with the avatar (*n* = 57, 92%) (Figure 3; See Table S1 for more details).

Older adults gave higher ratings than the younger adults on 5 of the items: more life-like than a video or phone call, comfortable talking to the avatar, felt like talking to the real person, felt like the avatar paid close attention, and believed the avatar understood the participant. For 2 of the items, older adults rated the factors the same or lower as compared to the younger adults: the participant understood the avatar, and the avatar resembled the volunteer.

**Figure 4.**
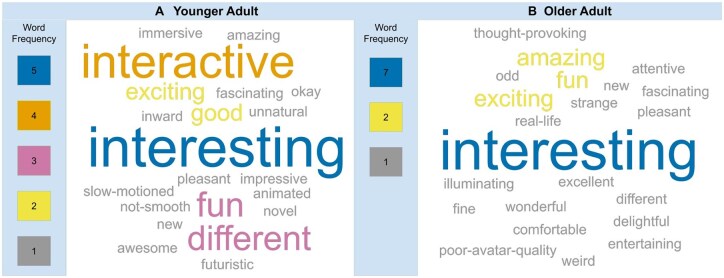
Word cloud generated in response to interaction with the volunteer’s 3D photorealistic avatar for younger adults (A) and older adults (B). Each word cloud highlights frequently mentioned words, with larger words representing higher frequency.

Figure 4 displays word clouds generated from the one-word perceptions of the experience reported by younger and older adults. Overall, participants from both age groups used positive words to describe their experience. *Interesting* and *exciting* were 2 words that occurred most frequently in both groups. *Interactive* was the second most frequently used word by younger adults, whereas no older adults used it to describe their experience. Some participants in both groups used descriptors conveying unpleasant or negative reactions, such as *weird*, *unnatural*, *slow-motioned*, and *strange*.

### Recognition of basic emotions conveyed by a 3D photorealistic avatar in HMD-AR

Summaries of the accuracy of the participants for both younger and older adults, for each emotion with both stimulus types (ie, video and avatar animations depicting emotions) are presented in Table 1. Overall identification of emotions displayed by the human video clips was high for both groups (>90%). Overall identification of emotions displayed by the avatar was 80% for the older adults and 93% for the younger adults.

**Table 1.** Older and younger adults’ accuracy in identification of emotions displayed nonverbally by humans in video clips vs by photorealistic avatars in head-mounted display augmented reality system (*N *= 1116 exposures/group).

Emotion	Older adults (*n* = 31)			Younger adults (*n* = 31)			Older vs younger adults	
	Correct identification		Video vs avatar	Correct identification		Video vs avatar	Human video clips (*n *= 558)	Photorealistic avatar (*n *= 558)
	Human video clips (*n *= 93/emotion)	Photorealistic avatars (*n *= 93/emotion)	Difference in accuracy, *p*	Human video clips (*n *= 93/emotion)	Photorealistic avatars (*n *= 93/emotion)	Difference in accuracy, *p*	Difference in accuracy (*p* value)	Difference in accuracy (*p* value)
Anger	96%	87%	.04	96%	98%	.61	96% vs 96% (1.00)	87% vs 98% (.01)
Disgust	88%	68%	<.001	97%	99%	.61	88% vs 97% (.05)	68% vs 99% (<.001)
Fear	75%	65%	.01	87%	84%	.44	75% vs 87% (.007)	65% vs 84% (<.001)
Happy	98%	77%	<.001	99%	94%	.20	98% vs 99% (.81)	77% vs 94% (<.001)
Sad	97%	95%	.61	100%	99%	.80	97% vs 100% (.46)	95% vs 99% (.33)
Surprise	91%	90%	.80	88%	82%	.12	91% vs 88% (.46)	90% vs 82% (.05)
Total	91%	80%	<.001	94%	93%	.25	91% vs 94% (.10)	80% vs 93% (< .001)

Each person was exposed to 3 exposures of each of the 6 emotions for human video clips (*n* = 18) and for photorealistic avatar (*n* = 18), resulting in 558 exposures/stimulus; 93 exposures/emotion. Statistically significant global interaction effect on accuracy of stimulus type and specific emotion with age group (*p* = .02). The specific comparisons shown in the table above are post hoc evaluations of the global interaction effect with an additional Bonferroni-corrected alpha.

In the overall mixed-effects model that included the 3 main effects of stimulus type (video, avatar), emotion (“anger,” disgust,” “fear,” “happy,” “sad,” and “surprise”), and age (younger adult, older adult), as well as each of the 2-way interaction effects and the overall 3-way interaction effect of the factors, there was a statistically significant interaction effect of all 3 factors on the accuracy with which emotions were perceived (likelihood-ratio chi-square_(5)_ = 14.10, *p* = .02). Each of the 2-way interaction effects with age group (stimulus type with age: likelihood-ratio chi-square_(1)_ = 12.65; emotion with age: likelihood-ratio chi-square_(5)_ = 47.62) was statistically significant (*p* < .001), and there was a statistically significant main effect of age group (likelihood-ratio chi-square_(1)_ = 19.46, *p* < .001). A sensitivity analysis using the aggregate percentage correct responses revealed very similar findings (3-way interaction: *F*_(5,300)_ = 2.52, *p =* .04; stimulus type with age: *F*_(1,60)_ = 14.13, *p* < .001; emotion with age: *F*_(1,60)_ = 7.31, *p* < .001; main effect of age group: *F*_(1,60)_ = 18.83, *p* < .001).

Across all 1116 exposures, younger adults identified emotions with greater accuracy than older adults (younger: 93.5% accurate; older: 85.6%; mean difference = 7.9%, 95% confidence interval [CI] = 4.3 to 11.4). Yet, because the differences varied considerably by stimulus and emotion (as revealed by the statistically significant 2-way interaction effects), we focus on those effects.

#### Interaction effect of age with stimulus condition

While overall, participants had greater accuracy in identifying emotions in the video stimulus condition than in the avatar condition (video: 92.7%; avatar: 86.4%, likelihood-ratio chi-square_(1)_ = 26.91, *p*  < .001), as noted by the significant interaction effect of stimulus with age group above, the difference in accuracy between those conditions was more pronounced in the older age group than in the younger group. Post hoc contrasts revealed that for the video stimulus, the difference in accuracy between the age groups was not statistically significantly different for the video stimulus (older: −3.6%, 95% CI = −7.9% to +1.7%, *p *= .10), yet it was for the avatar stimulus (older: −12.2%, 95% CI = −16.5% to −8.0%, likelihood-ratio chi-square_(1)_ = 31.89, *p*  < .001).

#### Interaction effect of age with type of emotion

Accuracy was highest for “sad” (97.5%), “anger” (94.1%), and “happy” (91.9%), while the lowest occurred with “fear” (77.7%), “disgust” and “surprise” (both 88.0%) (main effect of emotion: likelihood-ratio chi-square_(5)_ = 108.01, *p*  < .001). However, as revealed by previously documented significant 2-way interaction effect of age group with emotion, significant age differences in accuracy were observed for some emotions but not for others. Bonferroni-corrected post hoc tests revealed no significant age differences in accuracy for the 2 emotions with highest overall accuracy, “anger” (younger = 96.8%, older = 91.4%, likelihood-ratio chi-square_(1)_ = 2.75, *p*  = .10) and “sad” (younger = 99.5%, older = 95.7%, likelihood-ratio chi-square_(1)_ = 1.35, *p*  = .25), as well as the emotion with the lowest accuracy, “surprise” (younger = 84.9%, older = 90.9%, likelihood-ratio chi-square_(1)_ = 3.33, *p*  = .07). Older adults identified the remaining emotions considerably less accurately than did the younger adults: “disgust” (younger = 97.8%, older = 77.9%, likelihood-ratio chi-square_(1)_ = 37.67, *p*  < .001), “fear” (younger = 85.5%, older = 69.9%, likelihood-ratio chi-square_(1)_ = 23.14, *p *< .001), and “happy” (younger = 96.2%, older = 87.6%, likelihood-ratio chi-square_(1)_ = 7.04, *p*  = .008).

#### Age effects depended on both the stimulus and the specific emotion

As noted above, we observed a significant interaction effect among the 3 factors on the accuracy with which emotions were perceived. As shown in Table 1, the correct identification of “surprise” ranged from 82% to 91% over various conditions. “Surprise” was most often misidentified as “fear” in both stimulus conditions (*video*: older = 8/93 presentations [8.6%], younger = 10/93 [10.8%]; and *avatar*: older = 7/93 presentations [7.5%], younger = 12/93 [12.9%]) (see Figure S2A-D). Older adults were not only significantly less accurate than the younger adults in recognizing “disgust” in both the video and avatar conditions, but they were even less accurate when the stimulus was an avatar (67.7% vs 88.2%, likelihood-ratio chi-square_(1)_ = 50.44, *p *= .001). “Disgust” was often misclassified as “fear”, which was more common for older adults (video: 10/93 presentations [10.8%]; avatar 19/93 [20.4%]). Misidentification of “happy” as “disgust” was common among older adults for the avatar stimulus condition (15/93 presentations [16.1%]), and finally, “fear” was the least accurately identified emotion, regardless of age and stimulus condition (range = 65% to 87%). All participants identified “fear” less accurately in the avatar condition than in the video condition (74.2% vs 81.1%, likelihood-ratio chi-square_(1)_ = 5.57, *p *= .02). “Fear” was most often misidentified as “surprise” (*video*: older = 17/93 presentations [18.3%], younger = 10/93 [10.8%]; *avatar*: older = 17/93 presentations, [18.3%], younger = 7/93 presentations [7.5%]), and “disgust” (*video*: older = 6/93 presentations [6.5%]; *avatar*: older = 11/93 presentations [11.8%], younger = 7/93 [7.5%]).

## Discussion and implications

The effectiveness of any interactive communication modality (ICM) in enhancing social connection is dependent on its quality and characteristics, as well as on the individual’s attitudes and perceptions.^8^ We examined how younger and older adults would rate the social presence of HMD-AR using 3D photorealistic avatars created by easily accessible mobile applications. Unlike many AR studies,^31^ we required both the volunteer and the participant to wear HMDs for the interactions and examined 2 important facets of social presence: participants’ feelings of connectedness and the ability of the avatar technology to transmit nonverbal cues of emotions. Overall, both younger and older adults were overwhelmingly positive in their interactions with a volunteer’s 3D photorealistic avatar, and for the most part, they were able to identify the nonverbal emotions displayed by the 3D avatar.

Few differences existed between younger and older adults’ perceptions of the quality of the ICM. Indeed, most felt like they were speaking to a real person. Interestingly, older adults were more accepting of the AR telepresence than younger adults, further eroding the common misconception about older adults being less accepting of novel technology.^41^ A recent meta-analysis of older adults’ acceptance of technology found that perceived usefulness, perceived ease of use, and social influence were significant factors predicting adoption.^42^ Thus, future work should incorporate these factors in designing and implementing new technology for older adults and their families.

Participants were less positive about how well the photorealistic avatar resembled the volunteer and the extent to which the HMD-AR experience was more life-like than conversing by phone or video. Avatar representation is an important factor influencing the quality of HMD-AR communication, and high-realism avatars are especially important when considering communication partners.^31^^,^^43^ Indeed, several participants, both young and old, described the experience in negative terms, such as “weird” or “strange.” As the avatar design process advances in easily accessible software programs, negative reactions to the realism of the avatar are likely to decrease.

Detecting nonverbal expressions of emotion has a profound impact on social functioning. Our use of human video clips was an essential first step in establishing a baseline by which to compare accuracy in identifying emotions displayed by the matching 3D photorealistic avatar animations. First, our sample of independent living older adults had similar rates of accuracy to younger adults in identifying emotions expressed by the human video clips, with the exception of “fear.” Two separate meta-analyses have reported that older adults have mild reductions in recognizing some emotions, including fear.^33^^,^^44^ The high rates of accuracy may reflect our sample of older adults who were healthy, independent, community-residing older adults; others have reported that older adults with higher emotion recognition have preserved gray and white matter volumes in brain regions directly involved in emotional processing.^45^

When examining the 3D photorealistic avatars’ presentations, younger adults had ratings similar to those of the human video clips. Older adults, on the other hand, had a significant drop in overall accuracy from the avatar displays and distinct emotions compared to human video clips, demonstrating a trade-off in the efficiency of producing low-cost avatars to avatar quality. The information loss during facial expression and body gesture mapping from the real person to the AR avatar may have resulted in subtler cues by which to determine the emotions. Older adults may be unable to discriminate emotions given this loss of information.^44^^,^^46^^,^^47^ For example, older adults more commonly misclassified “disgust” with “happiness’” than did the younger adults. Since both emotions have a common prominent facial feature (parted lips), older adults may have relied more heavily on the mouth, ignoring subtler cues to make their choice.^47^ Alternatively, younger adults’ identification of avatar emotions may be a product of greater experience with virtual reality and avatars. In general, younger adults have more experience with VR-HMDs and avatars than older adults, although the extent to which VR experiences can be transferred to AR experiences is somewhat controversial.^31^ Last, older adults often rely on contextual factors, including language content and voice cues such as tone and volume.^48^ Our experiment did not allow for these factors, and it is likely that the addition of these factors to the experiment would have resulted in higher accuracy in detecting avatar emotions.

Our findings must be considered in light of the rapidly developing state of the science on immersive technologies, including the mapping of facial expressions and natural body motions of 3D photorealistic avatars. HMD-AR is among the newest immersive technologies, and even our sample of younger adults lacked experience with this technology. Research on AR avatars and their representations is in its early stages and typically does not include older adults. Various modalities exist for conveying emotions through realistic avatars, such as dedicated hardware to allow mapping of eye and facial movements to the avatar or algorithms that approximate facial motion by generating lip and eye movements.^24^ However, efficient reconstruction of 3D photorealistic avatars remains expensive in terms of high-cost capture systems and long capture processes.^18^ HMDs and smart glasses are also rapidly changing.^49^ For our study, we used the Microsoft HoloLens 2 headset, which is no longer available. As avatars, head mounted displays, and smart glasses become more sophisticated, older adults should be involved with not only the technical developments but also in the social aspects, adoption, and frequency and duration of use.^49^

In post hoc summaries, we observed an increase in the accuracy of the participants’ responses to the emotion classification task in response to the avatar during the first and second half of the presentation sequence, by 3.6% in younger adults and 8.1% in older adults. The difference was not statistically significant, and a “practice” effect cannot be conclusively stated given that accuracy varied considerably among emotions, which were not equally balanced in the first and second half cases. Yet, it raises an interesting question of whether the lower accuracy of older adults was due to less prior exposure to avatars. It may be that if older adults are given more time and exposure to interact with virtual avatars, their ability to detect emotions from avatars may improve.

### Limitations

Our study provides valuable insights into younger and older adults’ perceptions of HMD-AR with 3D photorealistic avatars as a tool for social connection. However, several limitations exist. First, we relied on questionnaires to gather data on their perceptions and attitudes. Future studies should incorporate additional measures, such as behavioral or physiological, and standardized questionnaires are needed. All 3 volunteers were young adults who identified as females. Our sample of younger adults consisted mainly of engineering students at the university and a higher proportion of men, while the older adults were active, independent living in the community, with a higher proportion of women. This sampling allowed us to compare older adults to a technologically savvy group of younger adults, but it does raise the question of whether the 3 volunteers unintentionally paid extra attention to the older adults, resulting in higher comfort and attention ratings. We did not include ­middle-aged adults, which further limits generalizability. Future studies could include a more diverse sample of older adults, such as those with cognitive and physical impairments or residing in long-term care settings. The evaluation was based on a short social interaction with a volunteer; future studies that focus on longer communications with family and friends may result in different perceptions of the realism and presence of the 3D photorealistic avatar. The study was conducted in the confines of the university’s engineering lab. Thus, we do not know the scalability of the technology in real-world settings, such as available internet bandwidth.

### Conclusion and future work

We demonstrated the viability of off-the-shelf creation of 3D photorealistic avatars as a means of social connection for older adults. Future work needs to focus on cohesive theoretical frameworks for AR communication that encompass multiple disciplines, such as engineering, nursing, medicine, psychiatry, and media psychology, that allow for the standardization of definitions and measures. To enhance generalizability, future work in the technical aspects must include older adults with varying cognitive and physical function, as well as other populations with diverse needs and capabilities. Ethical considerations are complex and need consideration in the use of HMD-AR as a social connection, such as intrusive advertising and surveillance from companies, manipulation of users’ perceptions and behaviors, loss of privacy, and gathering personal data without consent or knowledge. Studies on enhancing collaborative activities between 2 or more people would help determine ways to increase social connections. Lastly, clinical trials on the efficacy and effectiveness of HMD-AR in reducing loneliness and social isolation among older adults by enhancing social connection with loved ones are needed. Advances in avatar rendering technology, including codec avatars^15^ and Gaussian splatting from mobile devices,^50^ will help in designing more realistic avatars with smoother movements. As technology becomes more affordable and available, and demonstrates effectiveness, implementation studies are necessary, especially in long-term care settings. For those older adults requiring greater assistance with technology, human resource issues will need to be addressed.

## Supplementary Material

igaf083_Supplementary_Data

## Data Availability

Data will be made available upon reasonable request of the author. Given the exploratory nature of this work, our study protocol and analysis plan were not preregistered.
