# Supplementary material for: Older and younger adults’ perceptions of augmented reality photorealistic avatars as a viable medium for interpersonal communication
Source: Innov Aging. 2025 Aug 9;9(9):igaf083. doi: 10.1093/geroni/igaf083 (PMC12505127; doi:10.1093/geroni/igaf083)
Supplement: igaf083_Supplementary_Data [file igaf083_supplementary_data.zip › innage suppl Tauseef et al.docx]

***Innovation in Aging* Supplementary Material:** **Tauseef et al. Older and Younger Adults’ Perceptions of Augmented Reality Photorealistic Avatars as a Viable Medium for Interpersonal Communication.**

**Supplementary Method**

*Avatar Generation*

We generated avatars of three female volunteers using a mobile-based software, [In3D.io, with a phone camera.](https://in3d.io/) To generate the avatar’s facial features, the volunteers were instructed to turn their heads in three directions- left, right, and up - while holding the mobile device in front of them. To generate the avatar’s body, the mobile device was kept stationary with a complete view of the volunteers, who were then asked to rotate 360 degrees slowly. These avatars were generated in the .FBX file format. The software took about 5 minutes to generate each avatar. Facial muscles were represented by blendshapes, a technique often used in 3D animation to create facial expressions that deform a mesh to create a specific look or appearance. The avatar body included a skeleton, bones, and muscles. Supplementary Figure 1 shows the photorealistic avatars of the volunteers used in the study.

*Facial and Body Data Transfer*

To map the volunteer’s body gestures and facial expressions onto their avatars, Azure Kinect and an iPhone 12 were used to collect the body pose data and blendshape values, respectively. We used [Unity Face Capture,](https://apps.apple.com/us/app/unity-face-capture/id1544159771) an iPhone-based application capable of detecting a user’s facial expressions and outputting the respective blendshape values to capture the volunteers’ facial expressions. Similarly, we used Azure Kinect to obtain the rotation values for each joint. After retrieving these values in Unity on the volunteer’s end, these were sent via a local network to the participant’s end, parsed, and mapped onto the avatar.

**Supplementary Figure 1.** Photorealistic avatars of three volunteers created with In3D.io


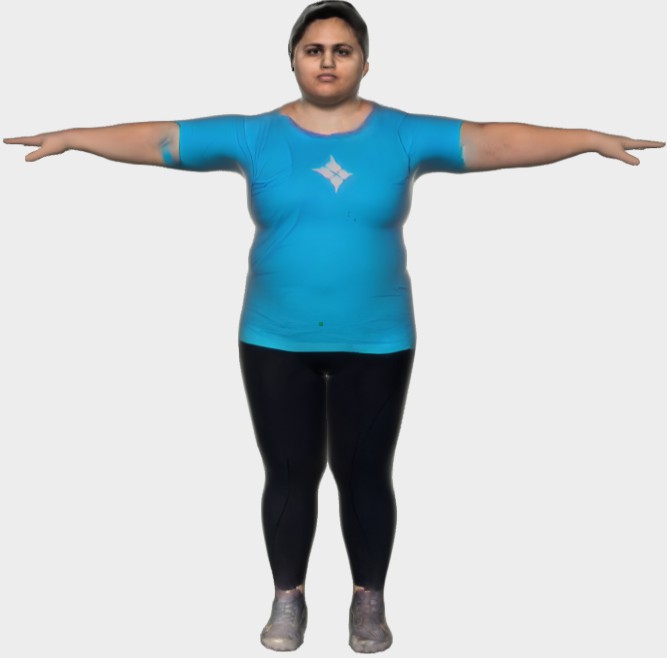

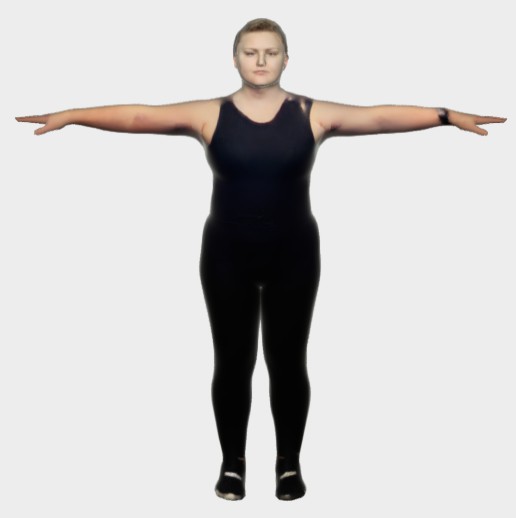

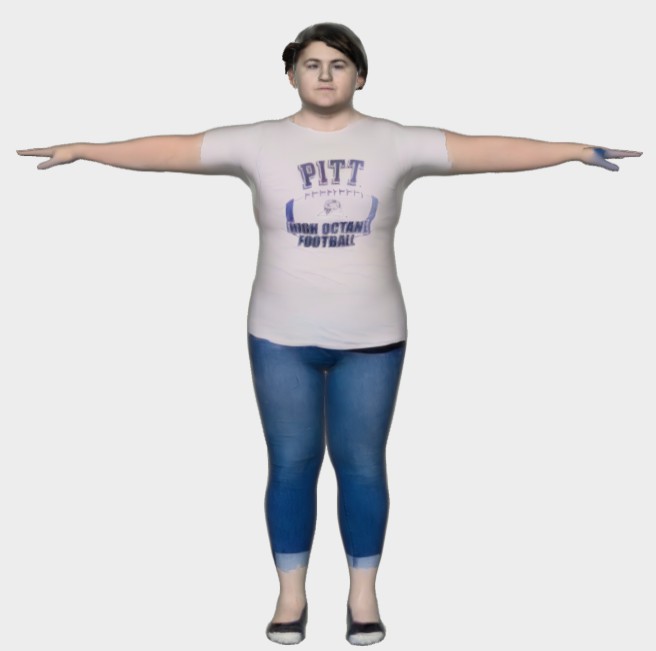


*Identification of Basic Emotions*

Comparisons of participants’ classification against the experts’ classifications of six basic emotions are depicted in the confusion matrix (Supplementary Figure 2). As shown in Table [1,](#_bookmark3) the correct identification of “surprise” ranged from 82 to 91% over various conditions. “Surprise” was most misidentified as “fear” in both stimulus conditions (video: 8/93 [older]; 10/93 [younger] and avatar: 7/93 [older]; 12/76 [younger]). “Disgust” was often misclassified as “fear”, which was more common for older adults (video: 10/93; avatar 19/93). Misidentification of “happy” as “disgust” was common amongst older adults for the avatar stimulus condition (15/93). Finally, as noted above, “fear” was the least accurately identified emotion regardless of age and stimulus condition. All participants identified “fear” less accurately in the avatar condition than in the video condition (74.2% vs. 81.1%, *p* = 0.01). “Fear” was most often misidentified as “surprise” (video: 17/93 times [older] and 10/93 [younger]; avatar: 17/93 times [older] and 7/93 [younger]) and “disgust” (video: 6/93 times [older]; avatar: 11/93 times [older] and 7/93 [younger]). **Supplementary Figure 2.** Confusion matrix depicting participants’ classification of emotions compared to an expert’s classification. Image **A** and Image **B** depict older adults’ classification in the video stimulus condition and avatar stimulus condition, respectively. Image **C** and Image **D** display younger adults’ classification in the video stimulus condition and avatar conditions, respectively.


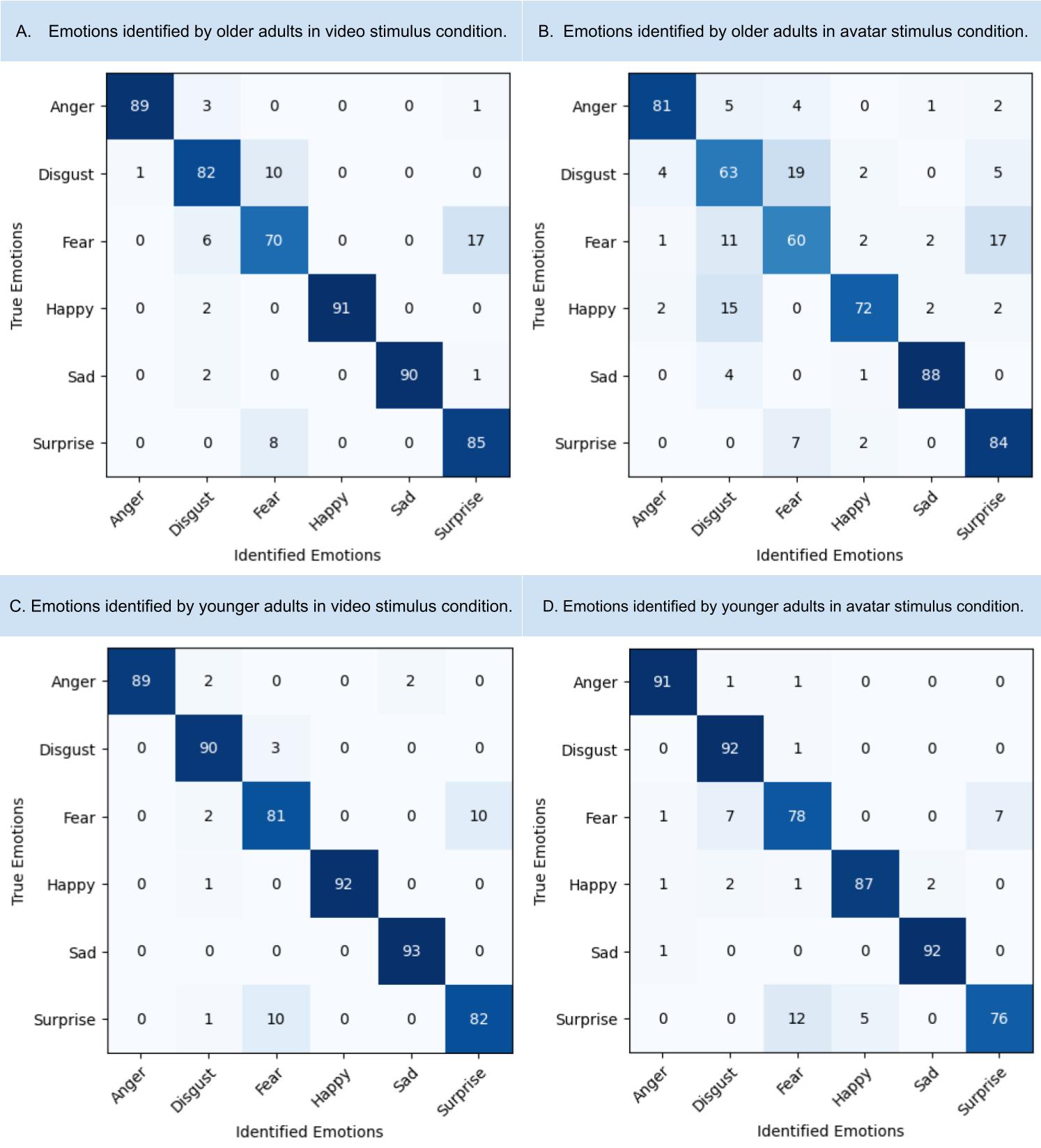


**Supplementary Figure 3.** Questionnaire on Ratings of 3D Avatar Interaction


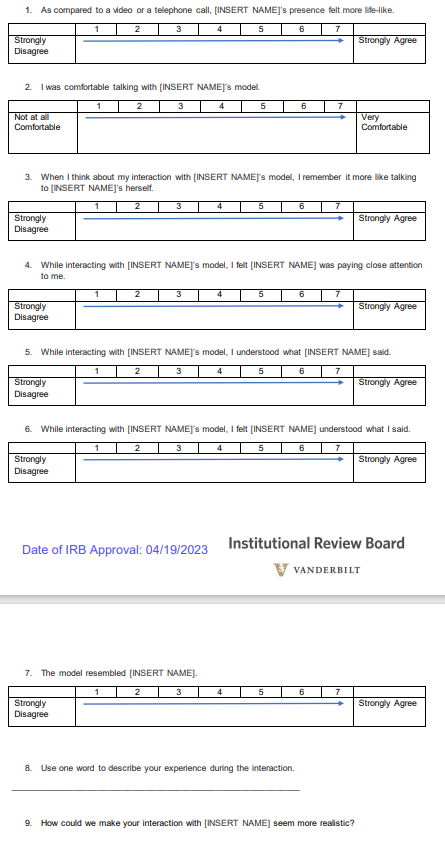


**Supplementary Table 1.** Participants’ Ratings of Quality of Interaction with a 3D Photorealistic Avatar

| **Item** | **All Participants**  **(n = 62)**  **N (%)** | **Young Adults**  **(n = 31)**  **N (%)** | **Older Adults**  **(n = 31)**  **N (%)** |
| --- | --- | --- | --- |
| *Item 1. Interacting with [INSERT NAME]’s model felt more life-like as compared to using a video or phone call to interact with someone.*  1. Strongly Disagree  2.  3  4.  5.  6.  7. Strongly Agree | 1 (2%)  8 (13%)  11 (18%)  7 (11%)  17 (27%)  9 (15%)  9 (15%) | 0  6 (19%)  6 (19%)  3 (10%)  9 (29%)  4 (13%)  3 (10%) | 1 (3%)  2 (6%)  5 (16%)  4 (13%)  8 (26%)  5 (16%)  6 (19%) |
| *Item 2. I was comfortable talking with [INSERT NAME]’s model.*  1. Strongly Disagree  2.  3.  4.  5.  6.  7. Strongly Agree | 0  1 (2%)  1 (2%)  3 (5%)  10 (16%)  15 (24%)  32 (52%) | 0  1 (3%)  1 (3%)  3 (10%)  7 (23%)  12 (39%)  7 (23%) | 0  0  0  0  3 (10%)  3 (10%)  25 (81%) |
| *Item 3. When I think about my interaction with [INSERT NAME]’s model, I remember it more like talking to [INSERT NAME] herself.*  1. Strongly Disagree  2.  3.  4.  5.  6.  7. Strongly Agree | 0  2 (3%)  7 (11%)  6 (10%)  18 (29%)  15 (24%)  14 (23%) | 0  1 (3%)  5 (16%)  3 (10%)  9 (29%)  9 (29%)  4 (13%) | 0  1 (3%)  2 (6%)  3 (10%)  9 (29%)  6 (10%)  10 (32%) |
| *Item 4. While interacting with [INSERT NAME]’s model, I felt [INSERT NAME] was paying close attention to me.*  1. Strongly Disagree  2.  3.  4.  5.  6.  7. Strongly Agree | 3 (5%)  1 (2%)  2 (3%)  3 (5%)  11 (18%)  9 (15%)  33 (53%) | 2 (6%)  1 (3%)  2 (6%)  3 (10%)  6 (19%)  7 (23%)  10 (32%) | 1 (3%)  0  0  0  5 (16%)  2 (6%)  23 (74%) |
| *Item 5. While interacting with [INSERT NAME]’s model, I understood what [INSERT NAME] said.*  1. Strongly Disagree  2.  3.  4.  5.  6.  7. Strongly Agree | 0  0  0  2 (3%)  2 (3%)  10 (16%)  48 (77%) | 0  0  0  1 (3%)  1 (3%)  7 (23%)  22 (71%) | 0  0  0  1 (3%)  1 (3%)  3 (10%)  26 (84%) |
| *Item 6. While interacting with [INSERT NAME]’s model, I felt [INSERT NAME] understood what I said.*  1. Strongly Disagree  2.  3.  4.  5.  6.  7. Strongly Agree | 0  0  2 (3%)  0  4 (6%)  10 (16%)  46 (74%) | 0  0  2 (6%)  0  4 (13%)  6 (19%)  19 (61%) | 0  0  0  0  0  4 (13%)  27 (87%) |
| *Item 7. [INSERT NAME]’s model resembled [INSERT NAME].*  1. Strongly Disagree  2.  3.  4.  5.  6.  7. Strongly Agree | 1 (2%)  3 (5%)  6 (10%)  12 (19%)  17 (27%)  16 (26%)  7 (11%) | 0  2 (6%)  2 (6%)  6 (19%)  9 (29%)  9 (29%)  3 (10%) | 1 (3%)  1 (3%)  4 (13%)  6 (19%)  8 (26%)  7 (23%)  4 (13%) |
